# Henry Johansson in memoriam

**DOI:** 10.1080/03009734.2017.1396271

**Published:** 2018-01-17

**Authors:** Lars Grimelius, Göran Åkerström

**Affiliations:** Uppsala University Hospital, Department of Pathology, Uppsala, Sweden; Uppsala University Hospital, Department of Surgical Sciences, Endocrine Surgery, Uppsala, Sweden


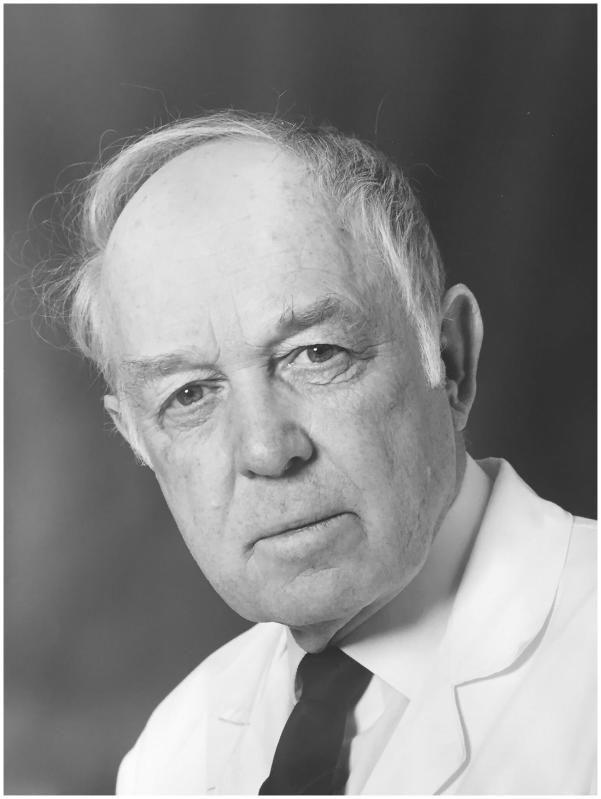


Henry Johansson (1929–2017), member of the board of the Upsala Medical Society for many years and a frequent contributor to this journal, has recently passed away. He studied medicine at the Karolinska Institute in Stockholm and then had a broad surgical training at the county hospital in Norrköping before he was employed at the Surgical Clinic at the University (Akademiska) Hospital in Uppsala in 1962.

Besides his clinical work he was very much involved in research. For his doctoral thesis he studied the influence of the thyroid function on intestinal motility in rats. This experimental model was later used by many younger colleagues in their research projects. When there was a subspecialization within the surgery discipline at the University Hospital Henry initially showed a special interest in oncologic surgery. Later on he changed to endocrine surgery, and he focused very much on the thyroid and parathyroid glands. When endocrine surgery finally became a subunit in General Surgery he became its first professor in 1985.

In his clinical research Henry noticed that the disease primary hyperparathyroidism (pHPT) was more common than previously recognized and often required surgery. In collaboration between the Departments of Surgery, Pathology, Histology, and Medicine he became the obvious leader of the basic studies concerning these glands and pHPT. For instance, he pointed out the connection between pHPT, psychiatric symptoms, and chronic renal failure. Together with Henry we carried out dissection studies of parathyroid glands in a comprehensive autopsy material. Methods were then developed to evaluate the functional activity of the parathyroid glands by parenchymal cell mass determination in different age groups of the human population (1).

Henry was a member of the Medical Faculty Board, and he was its vice-dean from 1987 to 1993. Together with Rector Magnificus of the University at that time, Professor Martin Henriksson Holmdahl (2), he promoted a further successful development of the surgical disciplines within the faculty. He belonged to the Upsala Läkareförening (Upsala Medical Society) for more than 40 years, and was its vice-chairman for several years. He was responsible for the annual evaluation of the doctoral dissertations of the Medical Faculty and the decisions on the Hwasser Award. Henry was an appreciated member of the National Patient Injuries Board. An article in our journal (3) testified to the scientific approach he had in this important task. Due to his broad research competence he served on the board of several research foundations.

Henry was strongly engaged in the education of refugee colleagues and their introduction to the Swedish Health Care system and research. He was also much involved in the Medical History Museum and the Medical History Association in Uppsala. His contribution in our journal on Ivar Sandström (4) has been much high-lighted because Sandström’s comparative studies of the parathyroid glands have been regarded as the last important anatomical discovery (5). Sandström’s paper was published in *Upsala Journal of Medical Sciences* in 1880, at that time named *Upsala Läkareförenings Förhandlingar*.

Henry Johansson exerted great influence on Swedish health care and medical research. He was a well-recognized, skilled, and very much appreciated surgeon with strong empathy for his patients. He had many friends, and he was always there when someone in his surroundings needed help and support.
